# Role of the Endocannabinoid System in Metabolic Control Processes and in the Pathogenesis of Metabolic Syndrome: An Update

**DOI:** 10.3390/biomedicines11020306

**Published:** 2023-01-21

**Authors:** Gabriella Dörnyei, Zsolt Vass, Csilla Berta Juhász, György L. Nádasy, László Hunyady, Mária Szekeres

**Affiliations:** 1 Department of Morphology and Physiology, Faculty of Health Sciences, Semmelweis University, Vas Street 17, 1088 Budapest, Hungary; 2 Department of Physiology, Faculty of Medicine, Semmelweis University, Tűzoltó Street 37-47, 1094 Budapest, Hungary; 3 Institute of Enzymology, Eötvös Loránd Research Network, Research Centre for Natural Sciences, Piarista Street 4, 1052 Budapest, Hungary

**Keywords:** endocannabinoid system, CB_1_ cannabinoid receptor, diabetes, metabolic disease, metabolic syndrome

## Abstract

Metabolic syndrome is a complex disease state, which appears mostly as a consequence of an unhealthy, sedentary lifestyle. Metabolic complications include insulin resistance (IR), diabetes, dyslipidemia, hypertension, and atherosclerosis, impairing life standards and reducing life expectancy. The endocannabinoid system (ECS) has an important role in signalization processes, not only in the central nervous system, but also in the peripheral tissues. Several physiological functions are affected, and overexpression or downregulation contributes to several diseases. A better understanding of the functions of cannabinoid (CB) receptors may propose potential therapeutic effects by influencing receptor signaling and enzymes involved in downstream pathways. In this review, we summarize recent information regarding the roles of the ECS and the CB_1_ receptor signaling in the physiology and pathophysiology of energy and metabolic homeostasis, in the development of obesity by enhancing food intake, upregulating energy balance and fat accumulation, increasing lipogenesis and glucose production, and impairing insulin sensitivity and secretion. By analyzing the roles of the ECS in physiological and pathophysiological mechanisms, we introduce some recently identified signaling pathways in the mechanism of the pathogenesis of metabolic syndrome. Our review emphasizes that the presence of such recently identified ECS signaling steps raises new therapeutic potential in the treatment of complex metabolic diseases such as diabetes, insulin resistance, obesity, and hypertension.

## 1. Introduction

Obesity and concomitant diseases such as diabetes, atherosclerosis, and other cardiovascular diseases (CVDs), as well as their consequences, now comprise a worldwide problem representing the highest entries in mortality statistics. The term metabolic syndrome has been introduced as a complex metabolic disorder accompanied by obesity and multiple cardiovascular risk factors for chronic diseases such as atherosclerosis, hypertension, and diabetes mellitus. The development of metabolic syndrome and metabolic pathologic disorders can be attributed to lifestyle, environmental, and genetic factors. A sedentary lifestyle and an unhealthy diet are the most obvious reasons [1,2,3,4,5,6,7,8].

It has been revealed that the endocannabinoid system (ECS) plays an important role in several physiologic regulatory mechanisms. Cannabinoid receptors were first identified in the nervous system as contributing to retrograde synaptic signaling [9,10]. Since then, the role of cannabinoid receptors has also been revealed in several tissues, such as in the cardiovascular, endocrine, and gastrointestinal systems [2,11]. Among their multiple roles, ECS and cannabinoid receptor signaling also play a regulatory role in food intake and energy metabolism. Activation of the cannabinoid type 1 receptor (CB_1_R) signaling pathway may upregulate food uptake, while inhibition of ECS signaling may depress food uptake mechanisms to develop weight loss [6,12,13]. Other studies have demonstrated a significant role of the ECS in lipid homeostasis [14,15,16]. Thus, investigation of the specific role of the ECS and cannabinoid signaling mechanisms can reveal important means for future therapeutic interventions.

Based on previous observations, in the present review, we aimed to summarize novel ECS signaling mechanisms with promising potential in the treatment of complex metabolic diseases such as diabetes, insulin resistance (IR), obesity, and hypertension.

## 2. Metabolic Syndrome

Metabolic syndrome, as a complex disorder, involves the disturbance of glucose metabolism, dyslipidemias, central obesity, and elevated blood pressure, promoting further cardiovascular morbidities such as atherosclerosis [2,3,4,5,6,7,8,17,18,19,20]. An earlier term has also been used, “syndrome X” or IR syndrome with hyperinsulinemia [6,17,21]. Clinical and metabolic studies have revealed the link between IR/hyperinsulinemia with dyslipidemia, elevations in triglyceride (TG) levels, reduced high-density lipoprotein (HDL), and elevated low-density lipoprotein (LDL) and total cholesterol levels. These in turn contribute to cardiovascular pathologies such as hypertension and atherosclerosis. Cardiovascular risk factors, which frequently occur in obesity and metabolic syndrome in populations of middle-aged adults independently of age, gender, ethnicity, and body mass index (BMI), include elevated TGs, low HDL cholesterol, and elevated LDL cholesterol [5,6,11,17,18,21]. According to previous observations, metabolic syndrome often involves the following components: high fasting glucose, IR with hyperinsulinemia, dyslipidemia, high blood pressure, and high BMI [3,5,6,19].

### Obesity and Insulin Resistance

Obesity often accompanies metabolic syndrome. An unhealthy diet (Western-type diet) in combination with a sedentary (inactive) lifestyle promotes the development of obesity and other symptoms of metabolic syndrome. The extent of obesity is often measured with BMI [1,5,6,11,22].

Obesity is often accompanied by IR, hyperinsulinemia, and dyslipidemia, which constitute further risks for CVDs. Neurohumoral activation also plays a role in the pathogenesis of obesity and metabolic syndrome through the production of adipokines such as adiponectin and leptin. Leptin produced by adipose tissue regulates hypothalamic appetite control mechanisms involving feeding behavior and hunger. Adiponectin is an anti-inflammatory hormone considered to be a protective factor against diabetes and IR. Obesity increases leptin levels and reduces adiponectin production, activating the renin–angiotensin system and inflammatory pathways such as TNF-α and NF-_K_B [6,23,24]. Visceral obesity, IR, and activation of neuroendocrine and inflammatory metabolites induce a metabolic inflammatory state, which leads to the development of complex metabolic disease with further cardiovascular risks [6,18,25]. The role of the opioid system in the development of obesity has also been described [26]. Infusions of beta-endorphins increased plasma levels of the pancreatic hormones insulin, C peptide, and glucagon and also elevated plasma glucose levels in young patients with obese relatives, which suggests the involvement of opioid peptides in metabolic events related to obesity [26].

Obesity and IR often develop into type 2 (non-insulin-dependent) diabetes (T2D). Patients diagnosed as type 2 diabetics are treated with antidiabetic drugs; in serious cases, they also may receive insulin. Among the prescriptions of treatment, it is important to improve patients’ lifestyle, maintain a special diet and perform regular activities. Type 2 diabetes (contrary to type 1) is often accompanied by obesity and metabolic syndrome. Thus, by organizing a healthy lifestyle and nutrition, progression of the disease and worsening of the condition of the patient can be prevented [3,6,14,15,18].

## 3. Endocannabinoid System and Its Physiological Roles

It is known that endogenously produced cannabinoids exert their physiological role by activating cannabinoid receptors. Cannabinoid receptors were originally named after their affinity for 9-tetrahydrocannabinol (THC), the main active ingredient of the extracts of *Cannabis sativa*. Endocannabinoids (eCBs) serve as endogenous ligands for cannabinoid receptors and participate in tissue-specific paracrine regulatory mechanisms first discovered in the nervous system [9,27]. The components of the ECS are the endocannabinoids, their receptors and intracellular signaling pathways, and enzymes that modulate their production and degradation [15,28,29,30,31]. The most important endocannabinoids can be listed as arachidonoyl ethanolamide (anandamide (AEA)), 2-arachidonoylglycerol (2-AG), noladin ether, O-arachidonoyl ethanolamine (virodhamine), and N-arachidonoyl dopamine [28,31,32,33]. AEA was isolated from pig brain tissue in 1992 by Devan [34]; later, 2-AG was discovered [35]. Production of 2-AG endocannabinoid is catalyzed by diacylglycerol (DAG) lipase (DAGL), and its degradation is due to monoacylglycerol (MAG) lipase (MAGL). Degradation of AEA is catalyzed by fatty acid amide hydrolase (FAAH) [11,31].

Endocannabinoids exert their actions on cannabinoid receptors. These include CB_1_Rs, which are characteristically present in neural tissues, and type 2 cannabinoid receptors (CB_2_R), which occur mostly in immune cells [9]. Cannabinoid receptors belong to the G-protein-coupled receptor (GPCR) family [28]. Cannabinoid-binding receptors were first characterized from brain tissue [34,36]. Since then, in addition to CB_1_Rs and CB_2_Rs, some other receptors have also been identified to respond to cannabinoid stimuli, such as GPR55 and TRPV_1_ [2,11,37]. CB_1_Rs are characteristically present in the central nervous system (CNS), typically with presynaptic neuronal location modulating the synaptic transmission [9,28]. During neuronal stimulation by neurotransmitters such as glutamate and acetylcholine, endocannabinoid-mediated CB_1_R presynaptic activation mediates the important function of depolarization-induced retrograde synaptic inhibition [9]. Signal transduction of CB_1_Rs is through heterotrimeric G proteins of the G_i/o_ type, inhibiting adenylyl cyclase and thus regulating calcium and potassium channels. In the case of depolarizing neurotransmitters (e.g., glutamate, acetylcholine), the concomitant release of endocannabinoids during cell signaling and the modulation of ionic channel activity will be an important neuromodulator action [30,38,39]. Several endocannabinoid compounds have been identified until now, including AEA and 2-AG [9,28,30]. 2-AG production has also been detected in the vascular smooth muscle cells of rat aorta [30,35]. AEA is a partial agonist of CB_1_Rs and has less affinity to CB_2_Rs, while 2-AG has been shown to have an affinity to both cannabinoid receptor subtypes [28,34,35]. CB_2_Rs have been found most of all on the immune cells and peripheral nerve endings controlling inflammatory and immune processes. They have been found to be located in the spleen, tonsils, and in hematopoietic tissues, as well as in cardiac and vascular tissues [2,11,32].

In addition to the earlier recognized key functions of endocannabinoids in the CNS, their roles in peripheral tissues have also raised interest [9,19,28,30,40,41,42]. It has been shown that the cannabinoid system plays a role in several important physiological mechanisms, in the field of cardiovascular, inflammatory, gastrointestinal, and endocrine regulations [19,28,30,40,43]. What is important now for us, their role in metabolic control processes, energy balance, and appetite regulation has been proven [44,45]. For a better understanding of the events in metabolic syndrome, we have to take into consideration the direct cardiovascular effects of endocannabinoids: negative inotropic, vasodilatator, and hypotensive actions have been reported [19,30,46,47]. With the growing number of studies on compounds modulating the ECS, new therapies for frequently combined metabolic–cardiovascular disorders appear on the horizon [11,43,46].

### Physiological Roles of CB_1_ Cannabinoid Receptors

CB_1_R was first discovered by its role in the CNS [36] and has been described to participate in retrograde synaptic signaling [9,10]. Later on, CB_1_Rs were detected in other tissues, such as in the peripheral nervous system, in the endothelium, and smooth muscle cells of vascular tissue; in fat tissue; in splanchnic organs; in the liver; and in skeletal muscle tissue as well [19,28,30,38,40,48]. There is growing evidence that endocannabinoids via CB_1_R play a role in a variety of physiological functions, such as maintenance of homeostasis and controlling the functions of several organs, such as vasoregulation, cardiac function, gastrointestinal and endocrine functions, energy metabolism, and appetite [19,28,30,40,43,46,47,49,50]. CB_1_R function can be activated by agonists and synthetic agonists, such as THC, WIN 55212, and HU210, and inhibition can be achieved by selective CB_1_R antagonists such as SR141716 (rimonabant), inverse agonist AM251, or neutral antagonist O2050 [11,28,30,40,47,49,51]. Activation of CB_1_R produces acute and chronic effects in tissues. In the cardiovascular system, vasodilation and cardiac depression with hypotensive action have been observed [11,49]. It has been reported previously in cell-expressing systems that activation of certain GPCRs such as the type 1 angiotensin receptor (AT_1_R) and calcium signaling may activate the DAGL enzyme to release 2-AG, which further mediates the paracrine transactivation of CB_1_Rs [40,42]. Angiotensin II (Ang II)-induced CB_1_R coactivation was inhibited by inhibitors of DAGL, which suggests that DAG generated from phosphoinositides during the AT_1_R signaling pathway is converted to 2-AG by DAGL [41,42]. In concert with this observation, we have found that in vascular tissue, Ang II-induced vasoconstriction is augmented by inhibition of CB_1_R and also of DAGL, whereas it is attenuated by inhibition of MAGL, suggesting that locally produced 2-AG activates vascular CB_1_Rs attenuating Ang II-induced vasoconstriction [30]. In recent studies, we have also found that endocannabinoid signaling moderates the tone of coronary arterioles [47,49]. CB_1_R signaling mechanisms due to locally released endocannabinoids are also involved in several metabolic processes, such as lipogenesis and altered glucose homeostasis. CB_1_R-induced lipogenesis is augmented in adipose tissue by stimulation of TG synthesis in the liver. Activation of CB_1_R-signaling also augments plasma TG and total cholesterol levels with depression of HDL. Related to carbohydrate homeostasis, CB_1_R activation can lead to gluconeogenesis, IR, and impaired glucose tolerance [2,11,15,52].

## 4. Role of Endocannabinoid System in the Metabolic Control Processes

The ECS has a significant role in several metabolic control processes. The ECS is present in the CNS, affecting appetite, food consumption, eating motivation, and energy homeostasis. The ECS can influence feeding control both in the CNS and in the periphery by influencing cell signaling pathways and the production and degradation of hormones and enzymes. The ECS promotes energy intake and storage, which favors overnutrition and the development of obesity and metabolic syndrome. The ECS and overactive cannabinoid CB_1_R signaling promote overnutrition, increases lipogenesis, and the risk of obesity and metabolic syndrome, including IR and dyslipidemia [14,15,16,18,20,28,43,44].

### 4.1. Effects of Endocannabinoid System on Fat Metabolism

The ECS influences fat metabolism by stimulating lipogenesis [11,14,16,28,53,54]. Activation of CB_1_R signaling stimulates lipogenesis and results in weight gain. Central CB_1_Rs located in the hypothalamus and limbic system are involved in the regulation of feeding. The ECS has important physiological regulatory functions not only in the CNS, but also in the periphery. Endocannabinoids and CB_1_R activation at the peripheral sites influence the metabolism of adipose tissue, liver, and skeletal muscle to promote lipogenesis. CB_1_Rs are expressed in both adipocytes and in hepatocytes; their activation increases lipogenesis while decreasing fatty acid oxidation in adipose tissue and the liver. Thus, endocannabinoids by peripheral CB_1_R activation contribute to diet-induced obesity and hepatic steatosis [11,28,51,54,55,56].

Endocannabinoid-induced lipogenesis involves several pathways. In hepatic tissue, endocannabinoids via the activation of CB_1_R increase expression of the lipogenic transcription factor SREBP-1c and its target genes such as acetyl-CoA carboxylase-1 and fatty acid synthase [55]. Endocannabinoids and CB_1_R activation also enhance preadipocyte maturation and trigger the peroxisome proliferator-activated receptor group, which in turn increases fat cell size and TG content by activation of TG synthesis from consumed fatty acids by inhibiting lipid breakdown and oxidation of fatty acids. Meanwhile, fatty-acid-synthesizing enzymes forming de novo fatty acids will be stimulated. Elevated 2-AG levels in skeletal muscle and adipose tissue cells activate CB_1_Rs and induce lipogenesis, but on the other hand, anti-lipogenic pathways will also be activated via TRPV_1_ receptors modulating visceral fat accumulation and adiponectin production [15]. Activation of CB_1_Rs by CB_1_R agonists increases lipogenesis in the liver also from non-fat-origin resources activating lipogenic enzymes in mice [56]. To prove the link between diet and the ECS, it was found that a high-fat diet (HFD) in mice increased endocannabinoid levels and expression of ECS enzymes in adipose tissue [57]. By detecting eCB levels in obese patients, it was found that although diet and obesity had no influence on eCB levels, expression of DAGL was upregulated, while mRNA expressions of MAGL and FAAH were downregulated in subcutaneous adipose tissue. However, interestingly, dietary fat intake reduced skeletal muscle CB_1_R and MAGL mRNA expressions, suggesting that a HFD influences ECS expression with tissue specificity [58].

### 4.2. Effects of Endocannabinoid System on Hunger and Appetite

Endocannabinoids and their receptors are involved at multiple levels in the control of energy homeostasis, food intake, and appetite by stimulating orexigenic pathways in the hypothalamus [6,11,51]. Endocannabinoids are orexigenic mediators and are part of the leptin-regulated central neural circuits that control energy intake [51]. Endocannabinoids via CB_1_R activation modulate the activity of hypothalamic neurons and the release of orexigenic and anorexigenic neuropeptides regulating energy metabolism to stimulate hunger [6,16,50,59,60]. These actions are mediated partly by leptin and ghrelin pathways, which modulate hypothalamic eCB levels. These pathways become deregulated during obesity with elevated hypothalamic eCB tone [6,16,61,62].

In some countries, cannabis-based drugs were used for improving appetite, mood, and ameliorating nausea in the 19th century. Cannabis-based therapy is still used nowadays for enhancing appetite or reducing nausea and pain in patients having chemotherapy and cancer [7,28,29,63]. CB_1_R activation increases appetite through actions on the CNS. A network of CB_1_R can be found in several nuclei of the hypothalamus, including the arcuate nucleus, paraventricular nucleus (PVN), ventromedial hypothalamus (VMH), and dorsomedial hypothalamus. These areas and their pathways have an important role in regulating the body’s homeostasis and several neuroendocrine functions [13,64]. To prove the direct link between cannabinoids and appetite control, it was observed in animal studies that injection of exogenous AEA (also an endocannabinoid) or THC into the VMH, or injection of AEA into PVN, significantly elevated the appetite of satiated animals [13,64].

Leptin is an important mediator in the control of food intake. Leptin, secreted by adipose tissue cells, is known to decrease food consumption by stimulating an anorexigenic pathway in the hypothalamus affecting the satiety–appetite system. Absence of leptin signaling elevated endocannabinoid levels in the hypothalamus, inducing hunger and overeating. After leptin treatment, hypothalamic AEA and 2-AG levels have been significantly reduced [16,61]. Defective leptin signaling is associated with elevated hypothalamic endocannabinoids in obese db/db and ob/ob mice and Zucker rats [61]. Leptin also stimulates the secretion and activation of FAAH, thereby decreasing the level of AEA. Leptin resistance has been found to reduce satiety, leading to obesity and secondary hyperleptinemia in several obese patients [45].

Components of the ECS located in the mesolimbic areas, the nucleus accumbens shell (NAcS) and the ventral tegmental area (VTA), participate in rewarding and motivational processes. Dopamine elicits a pleasure feeling in several places of the CNS; levels can also be enhanced by reward-related conditioning stimuli. Absence of CB_1_Rs reduces the stimulating effect on dopamine secretion, thereby preventing the development of addiction and rewarding traits [60]. The CB_1_R antagonist rimonabant inhibits dopamine secretion in the NAcS after food intake. However, in the NAcS, eCB levels increase during starvation and decrease during feeding. Dopamine regulates eCB levels with a negative feedback mechanism. The need to consume delicious food increases after food withdrawal, and a more rewarding sensation will be produced after food consumption. If an imbalance of the negative feedback occurs, an elevated ECS tone enhances food enjoyment resulting in hyperphagia [60].

Signals of gastric saturation enter the brainstem. After food intake, peptides such as ghrelin are released in the stomach, which is an important regulator peptide of appetite control. Another regulator peptide, cholecystokinin (CCK), is present in areas of the brain where nutrition and behavioral functions are regulated; in the cortical and limbic areas, it is coexpressed with CB_1_Rs. CCK reduces CB_1_R expression with a negative feedback mechanism, thereby reducing the number of CB_1_Rs after a meal. Food deprivation increases the number of CB_1_Rs, while the release of ghrelin attenuates the inhibition effect of CCK on CB_1_R. In the case of decreased CCK levels, the opioid signaling pathway is stimulated, which also has a major impact on the reward system [53]. The peripheral CB_1_R antagonist AM6545 in diet-induced obese mice induces an hypophagic effect. This can be reversed by inhibition of CCK receptors, indicating that obesity-associated hyperphagia is mediated by the mechanism including CB_1_R-mediated inhibition of gut–brain satiation signaling [44].

Motivational aspects of feeding include pathways involving the ghrelin-activated reward system in relation to dopamine, opioid, and endocannabinoid pathways [65]. Cannabinoid agonists stimulate the activity of VTA dopamine neurons, which enhances the release of dopamine in the NAcS, while antagonists of CB_1_R reduce dopamine release. Thus, ghrelin-activated dopamine release is regulated by the signaling processes of the ECS. Activation of the ECS promotes energy storage, generating fat accumulation and increasing caloric intake by stimulating appetite and the consumption of delicious food. In addition, administration of low-dose THC induces gluttony due to its appetite-stimulating effect. In experimental animals, after injecting AEA into the ventromedial nucleus of the hypothalamus, hyperphagia appears. In addition, the injection of endocannabinoids into the NAcS affected eating habits by reducing eating motivation [13].

### 4.3. Role of Endocannabinoid System in the Pathogenesis of Obesity

Obesity is a condition that contributes to the development of several metabolic comorbidities. In obesity, the balance between food intake and metabolism shifts, and excessive fat storage will be typical. General availability of cheap energy- and fat-rich diets available results in what can be called a pandemic of obesity. It has been proven that the levels of endocannabinoids, the number of cannabinoid receptors, and the availability of arachidonic and linoleic acids are all increased in obese patients. Linoleic acid is found in large amounts in the Western-type diet, which contains excessive fat, and it facilitates endocannabinoid synthesis. An overreaction of the ECS plays a role in the pathogenesis of obesity, IR, and atherosclerosis [2]. Activation of CB_1_Rs by endocannabinoids increases appetite, lipogenesis, and weight gain and results in obesity with metabolic complications [3,11,15,16,28,53,55]. The ECS plays a role in the regulation of energy turnover by stimulating both the CNS and peripheral nervous system to elevate food intake, fat storage, and lipogenesis, which in turn result in obesity and metabolic diseases [3,28,52]. It was found by Ruiz de Azua and Lutz that an increase in fat intake even without increasing caloric consumption in mice resulted in increased body weight compared to control mice fed on a conventional diet [16]. ECBs increase adiponectin secretion and fat storage by increasing the amount of newly produced fatty acids and TG synthesis in adipose tissue cells, while at the same time degradation of fatty acids is decreased. Activation of CB_1_Rs in the liver also increases the production of fatty acids, so increased plasma cannabinoid levels can lead to the formation of nonalcoholic liver steatosis. All the above-mentioned mechanisms contribute to obesity [3,11,14,15].

CB_1_R signal pathways activate orexigenic ones in the hypothalamus, stimulating appetite and promoting obesity [6,54,60]. At the periphery, cannabinoid CB_1_Rs are important participants in obesity-induced metabolic inflammation, IR, and dyslipidemia [18]. It was found that AEA administration into VMH increased food consumption and induced significant hyperphagia resulting in obesity, which was attenuated by pretreatment with selective CB_1_R antagonist SR141716 in rats [13]. Stimulation of CB_1_ cannabinoid receptors increased the amount of consumed food, and on the other hand, inhibiting the receptors resulted in an improvement of insulin sensitivity and also decreased body weight and improved metabolic parameters [6,11,28]. Further, to verify the role of CB_1_R in obesity and metabolic processes, CB_1_R knockout (CB_1_R-KO) mice have been applied [66]. The role of CB_1_Rs in appetite and weight gain could be verified by the fact that CB_1_R-KO mice had a more moderate risk of obesity and weighed less than their wild-type counterparts, which could be attributed to a mild deficit in adipose tissue mass [28,53,67]. It is even more important that CB_1_R-KO mice were resistant to fatty-diet-induced obesity with similar caloric inputs to their wild-type mates. This suggests the presence of an additional peripheral CB_1_R contribution to the development of obesity. CB_1_R-KO mice are also resistant to obesity-accompanied changes in metabolic parameters, including hyperlipidemia and elevated plasma insulin and leptin levels that consequently appear in obese wild-type (WT) mice. CB_1_R-KO mice have lower leptin levels and enhanced sensitivity to the anorectic effect of leptin [28,55,67]. These metabolic changes observed in CB_1_R-KO mice could also be initiated by treatment with CB_1_R antagonist SR141716 [67,68].

In obesity, ECS and CB_1_Rs are also characteristically found upregulated in liver and adipose tissue. In WT mice, 3 weeks of a HFD increased hepatic fatty acid synthesis. These mice developed hepatic steatosis. This liver steatogenic effect of HFD was partly reversed by SR141716 [55]. These findings indicate that a HFD activates eCBs, which contributes to lipogenesis and hepatic steatosis and thus the development of obesity [28]. CB_1_R-dependent metabolic effects in relation to appetite and obesity are summarized in Table 1.

Since endogenous cannabinoids by acting on CB_1_Rs stimulate appetite and lipogenesis, CB_1_R antagonists seem to form a promising treatment for obesity. Treatment with CB_1_R antagonists (such as rimonabant) caused a decrease in food intake and produced a sustained weight loss in animals with diet-induced obesity [11,18,51,68]. In animal experiments, HFD-induced obesity could be prevented in the absence of CB_1_Rs in CB_1_R-KO mice. CB_1_R KO mice are detected to have less body weight, decreased body fat, and improved glucose homeostasis and plasma lipid profile compared to their wild-type mates [16]. The CB_1_R blockade also improves glucose tolerance, insulin and leptin sensitivity, and lipid profile in diet-induced or genetically obese animals [11,68]. CB_1_R-KO mice were resistant to HFD-induced hepatic steatosis. In addition, in HFD-fed WT mice, administration of the CB_1_R antagonist decreased fatty acid production in the liver [56].

Clinical trials with rimonabant involving obese patients with metabolic syndrome suggest beneficial effects of chronic CB_1_R blockade in reducing body weight and also in improving glucose tolerance and lipid profile. CB_1_R antagonist rimonabant also reduced plasma leptin and insulin levels, while it increased plasma adiponectin levels [11,69,70]. In a recent study, however, decreasing the level of 2-AG by inhibiting its degrading enzyme MAGL attenuated HFD-induced obesity. Surprisingly, MAGL-deficient mice fed a HFD gained less body weight than wild-type mice and were also protected from IR and hepatic steatosis. Experiments on double MAGL-CB_1_R-KO mice then indicated that these mechanisms could be independent of CB_1_R signaling, suggesting other functions of the enzyme [71].

### 4.4. Role of Endocannabinoid System in the Pathogenesis of Insulin Resistance

IR is a disorder of the carbohydrate metabolism, an important component of the metabolic syndrome family, often called the “ante-room of T2D”. In IR, by decreased cellular insulin sensitivity, metabolic regulation of glucose homeostasis is damaged in peripheral tissues and blood. According to novel data, we can state that the ECS has an important role in the regulation of insulin signaling pathways. Activation of CB_1_Rs stimulates appetite, metabolic disorders such as lipogenesis, dyslipidemia, and obesity, and disorders of carbohydrate metabolism developing IR and T2D [52].

Endocannabinoids are orexigenic hormones, so an elevated ECS tone increases appetite and food intake, resulting in obesity, which is a major risk factor in developing IR and T2D. AEA via CB_1_R increases the craving for delicious meals and enhancement of energy storage, but this effect could not be observed in CB_1_R-KO mice. High levels of AEA and 2-AG are associated with IR, elevated levels of visceral fat, and dyslipidemia [33].

During obesity, dysregulation of the ECS contributes to visceral fat accumulation and suppresses the synthesis of adiponectin, decreasing insulin sensitivity and fatty acid oxidation. Thus, further several cardiometabolic risk factors may develop that are associated with T2D and obesity [15]. In the skeletal muscle, eCBs disrupt the insulin signaling pathway, and stimulation of CB_1_Rs in the liver depresses insulin sensitivity and insulin production. In addition, eCB signaling excites endoplasmic reticulum stress levels, which increases the levels of long-chain ceramides in the liver, inhibiting insulin signaling [52]. In patients with T2D, higher circulating levels of endocannabinoids, as well as AEA or other eCBs, can be detected than in patients without diabetes with similar body mass [33,72,73]. This observation was further supported by decreased endocannabinoid levels measured during successful therapeutic (dietary) interventions to induce weight loss and improve insulin sensitivity [33,74,75].

Stimulating CB_1_R with specific agonists increases the secretion of insulin, somatostatin, and glucagon; it increases fat storage by stimulating lipoprotein lipase and release of adiponectin, and as a result, hepatic steatosis and IR develop. CB_1_R-induced activation inhibits insulin signaling mechanisms by inhibiting insulin receptor substrate-1 and protein kinase B (AKT) phosphorylation, depressing pancreatic beta cell function, which mechanisms contribute to IR. Conversely, lack of CB_1_Rs or inhibition of CB_1_Rs improves insulin signaling, thus improving IR and pancreatic beta cell function and reducing hepatic steatosis and obesity [52,76,77]. In addition, in the mouse beta cell line and in human islets, CB_1_R agonists diminished insulin secretion, whereas silencing CB_1_Rs in the beta cell line increased the expression of proinsulin, glucokinase, and glucose transporter 2 (GLUT2), which was also observed in the beta cells of CB_1_R-KO mice [78]. On the other hand, in adipocytes, it was found that activation of CB_1_R by 2-AG promoted insulin sensitivity by increasing insulin-stimulated AKT phosphorylation, which was attenuated by the CB_1_R antagonist. This mechanism may serve CB_1_R-dependent lipid accumulation [79]. Targeting ECS-dependent lipid signaling in the peripheral tissues can be a potential therapeutic means to treat IR and T2D. Applying CB_1_R antagonists or inverse agonists as adjuvant therapy to lifestyle modulation by weight reduction, exercise, and glycemic and lipemic control in obese and T2D patients seems to be beneficial [15,33,52]. CB_1_R-dependent metabolic effects in relation to IR and T2D are summarized in Table 2.

### 4.5. Role of Endocannabinoid System in Hypertension

The endocannabinoid system is involved in the regulation of cardiovascular function. Complex mechanisms of cardiovascular effects of cannabinoids involve modulation of autonomic outflow in both central and peripheral nervous systems and also the direct effects on myocardium and vasculature. CB_1_R-induced effects have been reported to exert vasodilatory, negative inotropic, and hypotensive actions [11,19,30,38,49,50]. Though CB_1_R stimulation exerts vasodilatory action, in CB_1_R-KO mice, blood pressure and heart rate are described to be normal [38,50,80]. Elevated blood pressure was observed to be effectively reduced by elevating eCB levels in a hypertensive rat model; however, systemic cannabinoids induced only a mild hypotensive action in normotensive animals [46,50]. We previously observed that inhibition of CB_1_R by O2050 augmented Ang II-induced vasoconstriction in wild-type mice, whose effect was not observed in CB_1_R-KO mates. In addition, acute Ang II infusion-induced pressure rise was further augmented with CB_1_R inhibitor O2050 in vivo in mice containing CB_1_Rs, which suggests a role of CB_1_Rs in the control of vascular tone and blood pressure [30]. Previously, in a human study, THC was demonstrated to reduce blood pressure [81], while in marijuana users, a higher prevalence of elevated blood pressure has been reported [50,82]. Though CB_1_R stimulation has been shown to be beneficial in hypertensive animal models [46,50], CB_1_R antagonism improved cardiac function after experimental myocardial infarction and metabolic syndrome [50,83]. In addition, elevated levels of CB_1_R and eCB tone may be beneficial in CVD, or it may also be an adaptive compensatory mechanism [50]. Thus, the role of the ECS in blood pressure regulation and its therapeutic potential in hypertension still need further clarification.

### 4.6. Summary of the Role of Endocannabinoid System in Metabolic Control Processes

**Figure 1:**
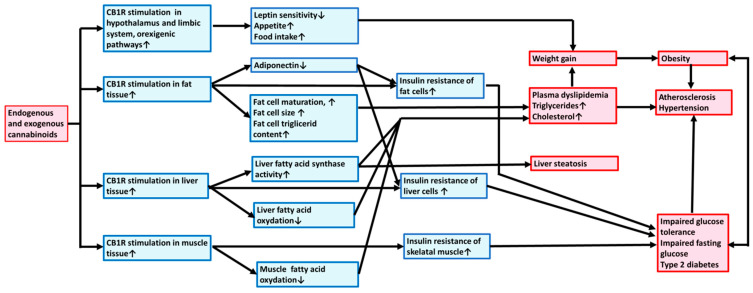
Exogenous and endogenous cannabinoids promote metabolic syndrome and atherosclerosis through central and peripheral type 1 cannabinoid receptors. Mechanism of action. Exogenous and endogenous cannabinoids via stimulation of CB_1_Rs activate orexigenic pathway, increasing appetite and stimulating fat, liver, and muscle tissues to promote lipogenesis, weight gain, and obesity. CB_1_R activation also induces alteration of lipid homeostasis elevating plasma triglyceride and cholesterol levels and thus increasing the risk for the development of atherogenesis, hypertension, and liver steatosis. In addition, CB_1_R activation by developing insulin resistance and gluconeogenesis may induce the risk for type 2 diabetes mellitus. CB_1_R, type 1 cannabinoid receptor.

The mechanisms leading to metabolic syndrome via the activation/overactivation of CB_1_Rs are summarized in Figure 1. Exogenous and endogenous cannabinoids via stimulation of CB_1_Rs activate hypothalamic orexigenic pathways, increasing appetite; promoting fat, liver, and muscle tissue lipogenesis and energy uptake; body weight elevation; and obesity development. CB_1_R activation also induces alteration of lipid homeostasis, elevating TG and plasma cholesterol levels and thus increasing the risk for the development of atherogenesis, hypertension, and liver steatosis. In addition, CB_1_R activation may induce the risk of T2D by developing IR and stimulating gluconeogenesis.

Increased expression of the components of the ECS and elevated levels of endocannabinoids increase food intake and produce hunger due to the activation of orexigen pathways in the hypothalamus. Several brain regions are involved in regulating food intake and are upregulated by the ECS. In addition, reward-related stimuli are conditioned by endocannabinoids in some brain regions. The ECS increases the anabolic processes; tonic enhancement causes hyperphagia, reduces energy expenditure, and increases glucose uptake and lipogenesis. It also suppresses the production of adiponectin, decreasing insulin sensitivity and fat oxidation [52]. Furthermore, CB_1_R activation enhances fat cell maturation, increases adipose storage capacity, stimulates fatty acid and TG synthesis, and inhibits fatty acid oxidation. Activation of CB_1_Rs in the liver can cause nonalcoholic hepatic steatosis with increased fat production [15]. All these mechanisms contribute to the development of obesity and worsen existing overweight. Inhibition of CB_1_R activity improves the peripheral lipid profile and may start recovery from metabolic syndrome by decreasing body weight and appetite, thus also improving glucose and lipid homeostasis and preventing atherosclerosis [15,20,52].

## 5. Therapeutic Potential of Endocannabinoid System in Complex Diseases of Metabolic Syndrome

Recently, the therapeutic application of cannabidiol, a cannabinoid derivative supposedly without central actions, has been raised and is under investigation.

Cannabinoids and endocannabinoids have been shown to play a role in modulating pathological conditions in inflammatory, neurodegenerative, gastrointestinal, metabolic, and cardiovascular diseases and in cancer. Cannabidiol-based drugs may be used for therapy of several pathological situations, such as pain, sleep disorders, neurodegenerative and psychiatric diseases, etc. [7,11,12,28,29,37]. We can state that modulating the ECS may have and will have therapeutic potential. Targeting the ECS may provide a novel option for the management of obesity and obesity-related diseases, type 2 diabetes, and several CVDs as well [3,14,20,28,29,51]. The sites of pharmacological interventions can be most of all the modulation of CB_1_R activity and signaling, as well as the modulation of enzymes responsible for the synthesis and degradation of endocannabinoids, especially FAAH and MAGL. Inhibition of FAAH and MAGL elevates endocannabinoid levels [11,46]. In addition, inhibition of MAGL or FAAH in vivo or in vitro may exert vasodilatory and hypotensive actions [30,46,47]. CB_1_R stimulation by its vasodilatory action may be beneficial in hypertension. Thus, CB_1_R agonism or elevation of eCB levels may exert certain therapeutic effects, such as antinociceptive, anti-inflammatory, vasodilatator, hypotensive, and anticancer actions [11,29,46].

Under normal conditions, the production of leptin increases the activity and amount of FAAH, thereby reducing endocannabinoid levels in the body. FAAH is responsible for breaking down AEA and therefore suppresses food intake. Upon suppression/inhibition of the FAAH or MAGL enzymes, endocannabinoid levels are elevated, and hyperphagia, leptin resistance, and obesity may develop. Suppression or absence of endocannabinoid-degrading enzymes is clearly associated with an increased risk of obesity [45].

As we could see earlier, CB_1_R has a crucial role in obesity-induced proinflammation and metabolic syndrome, including IR and dyslipidemia. Targeting the receptors this way can be a promising therapeutic strategy in obesity and metabolic syndrome. Inhibition of the CB_1_R may have a beneficial effect in the prevention and treatment of metabolic syndrome, improving glucose homeostasis and IR [11,18,51]. CB_1_R antagonists successfully targeted obesity-induced metabolic disease; among them, rimonabant was proven to be a promising treatment in obesity to induce weight loss and improve dyslipidemia [11,25,51,69,70]. Rimonabant has been subjected to several clinical trials, including patients being obese, overweight, dyslipidemic, or suffering from hypertension or metabolic syndrome and type 2 diabetes. Rimonabant medication significantly decreased body weight and hip circumference, while it increased HDL levels and reduced LDL cholesterol, TG, and HbA1c levels in obese patients in contrast to the control group. Rimonabant, by its actions of reducing body weight and improving dyslipidemia and glucose homeostasis, significantly reduced the chance of having CVDs and metabolic syndrome. However, bad mood and nausea were the most common side effects. In several cases, even withdrawal of the therapy has been implemented because of its inconvenient psychotic side effects. Development of medication with specific therapeutic effects and with fewer side effects is now the focus of pharmacological research [6,11,25,37,51,69,70,84].

The beneficial actions of CB_1_R antagonism have been further investigated with new generations of CB_1_R antagonists. Since increased tissue fibrosis may accompany CB_1_R activity, due to an interplay between the ECS and inflammatory mechanisms, the antifibrotic efficacy of CB_1_R antagonism can form a new therapeutic potential. Second- or third-generation CB_1_R antagonists may have therapeutic potential in pulmonary or liver fibrosis [12]. In animal experiments, the development of diabetes-induced cardiomyopathy and fibrosis has been attenuated and prevented by treatment with the CB_1_R antagonist, which was observed also in CB_1_R-KO mice [85]. Similarly to CB_1_R inhibition, a decrease in eCB levels by inhibition of DAG lipase may be beneficial in some chronic diseases, such as neurodegenerative and metabolic disorders [86].

On the other hand, the therapeutic potential of FAAH inhibition is still controversial. A previous pharmacology model showed that complete inhibition of FAAH was insufficient to raise the endogenous ligands enough to produce significantly increased pharmacological activity [87]. Pharmacodynamic and pharmacokinetic studies have been performed on different FAAH inhibitor components (e.g., BIA 10-2474, PF-04457845, and JNJ-42165279). BIA 10-2474 was a less potent FAAH inhibitor than PF-04457845 in humans, but it was effective in mouse FAAH enzymes [88]. Although BIA 10-2474 was released to clinical trials, it has been retrieved due to its serious side effects [87,88].

To summarize ECS-related therapeutic potential, it is suggested that moderate activation of CB_1_Rs by selective agonists or by endocannabinoids, as well as the elevation of eCB levels by inhibition of degrading enzymes MAGL or FAAH, may have acute beneficial therapeutic actions such as pain relief and antipsychotic effects, beneficial outcomes in some neuropsychiatric diseases, and potential beneficial effects in hypertension by a CB_1_R-dependent vasodilatory effect.

However, long-term antagonism of CB_1_Rs has been proven to be beneficial in obesity-related disorders, improving glucose and lipid homeostasis and inducing weight loss. In addition, CB_1_R antagonism has been shown to be beneficial in the prevention of chronic inflammation and fibrosis. Similar effects can be produced by inhibition of 2-AG-producing DAG lipase [3,6,11,12,14,20,28,29,37,43,86]. Recently, reports on new generations of CB_1_R antagonists have been published with limited neurobehavioral and psychiatric side effects [12,18]. CB_1_R antagonists/inverse agonists are potential beneficial adjuvants to lifestyle modification and weight reduction in the control of carbohydrate and lipid homeostasis to prevent dyslipidemia and hyperglycemia in obese and T2D patients.

We can conclude that this complex lipid signaling system can serve as a potential therapeutic source in metabolic syndrome, pathologic obesity, and even T2D [1,15,33]. Modulation of the ECS can serve as part of a complex therapy for obesity-related metabolic disorders, such as IR and diabetes mellitus [1,11,15,29].

## 6. Summary and Conclusions

In the present review, we summarized the role of the ECS and CB_1_R activation in energy homeostasis and metabolism and in the development of metabolic syndrome involving obesity, IR, type 2 diabetes, and dyslipidemia. The metabolic regulatory role of the ECS is manifested partly centrally through brain regions (mostly hypothalamic) controlling the nutritional and metabolic processes of the body with the involvement of neural CB_1_R activation influencing neuroendocrine functions. Central endocannabinoids increase appetite by stimulating orexigenic pathways, changing the homeostatic balance toward energy storage, and weight gain. Their direct and indirect peripheral actions increase glucose uptake and lipogenesis in adipose tissue and stimulate the de novo synthesis of fatty acids and glucose in the liver. Elevated endocannabinoid activation promotes obesity and obesity-linked disorders such as metabolic syndrome, glucose intolerance and type 2 diabetes, and dyslipidemia, with the subsequent risk of atherosclerosis and further CVDs. These ECS-dependent effects are attributed mainly to CB_1_R activation and its signaling mechanisms. Inhibition of CB_1_Rs has been shown to exert beneficial therapeutic effects improving metabolic conditions by decreasing obesity and inducing weight loss, improving glucose homeostasis and lipid profile, and reducing fibrosis in several chronic diseases of different parenchymal organs. Based on these observations, it can be stated that modulation of the ECS may provide novel therapeutic strategies, especially after developing a new generation of CB_1_R antagonists with limited psychologizing effects.

Thus, pharmacological modulation of the ECS forms a promising therapy for complex treatment of obesity-related metabolic diseases, IR, and diabetes mellitus. Modulation of eCB activity, using new-generation CB_1_R antagonists and also improvement of lifestyle factors, may serve as complex therapy for obesity-related metabolic disorders, such as IR and diabetes mellitus.

## Figures and Tables

**Table 1:** Effects of endocannabinoid system and cannabinoids on appetite and obesity. Analysis from references. Original and review articles discussing CB receptor-dependent methods (KO mice, agonists, antagonists) are emphasized. CBR, cannabinoid receptors, CB_1_R and CB_2_R, cannabinoid receptors type 1 and 2, CCK, cholecystokinin, KO, knockout, HFD, high-fat diet, eCB, endocannabinoid, ECS, endocannabinoid system, DAGL, diacylglycerol lipase, MAGL, monoacylglycerol lipase, FAAH, fatty acid amide hydrolase, 2-AG, 2-arachidonoylglycerol, THC, tetrahydrocannabinol, Glu, glutamatergic.

Ref.	Article Type	Animal Model	Diet	Receptors, Agonist/Antagonist	Short Summary	Studied Mechanism and Disease
Jamshidi and Taylor, 2001 [13]	Animal study	Rats		CBR agonist AEA, CB_1_R antagonist rimonabant	In presatiated rats, intrahypothalamic injection of AEA increases appetite by hypothalamic CB_1_R activation.	Appetite
Argueta, Perez, Makriyannis, and DiPatrizio, 2019 [44]	Animal study	Mice	High-fat diet and sucrose diet	CBR agonist WIN55212, peripheral CBR antagonist AM6545	AM6545 decreases appetite in diet-induced obesity mice, which is reversed by CCK receptor antagonist. The mechanism of hyperphagia-associated obesity includes CB_1_R-mediated inhibition of gut–brain satiety signaling.	Appetite, diet-induced obesity
Osei-Hyiaman et al., 2005 [55]	Animal study	CB_1_R-KO mice	High-fat diet	CBR agonist HU210, CBR antagonist SR141716 (rimonabant)	CB_1_R-KO mice are resistant to diet-induced obesity. CBR agonist increases hepatic fatty acid synthesis. HFD increases liver eCB levels.	Diet-induced obesity
Engeli et al., 2014 [58]	Clinical study		High-fat diet		HFD does not influence eCB levels, but tissue-specific DAGL is upregulated, and FAAH and MAGL are downregulated in obese patients. HFD reduces skeletal muscle CB_1_R and MAGL expression.	Obesity
Bellocchio et al., 2010 [59]	Animal study	Glu-CB_1_R-KO mice		CBR agonist THC	THC-inducedhyperphagia is completely blunted in Glu-CB_1_R-KO mice. Ventral striatal CB_1_Rs exert a hypophagic action through inhibition of GABAergic transmission.	Appetite, obesity, and obesity-related disorders
Di Marzo et al., 2001 [61]	Animal study	CB_1_R-KO mice, leptin-deficient ob/ob and db/db mice		CBR antagonist rimonabant	CB_1_R-KO mice eat less, and CB_1_R antagonist reduces food intake in wild-type mice. Defective leptin signaling elevates hypothalamic eCBs in obese ob/ob and db/db mice that show a hyperphagic phenotype.	Appetite, obesity, and obesity-related disorders
Ravinet Trillou, Delgorge, Menet, Arnone, and Soubrié, 2004 [67]	Animal study	CB_1_R-KO mice	High-fat diet	CBR antagonist SR141716 (rimonabant)	CB_1_R-KO mice are hypophagic with less body weight and do not develop obesity. CB_1_R inhibition reduces plasma insulin and leptin levels. CB_1_R activation is a key factor in diet-induced obesity.	Diet-induced obesity
Poirier et al., 2005 [68]	Animal study		High-fat diet	CBR antagonist rimonabant	Obese mice demonstrate abnormal serum lipid profile. Rimonabant treatment decreases body weight and improves lipid profile in obese mice.	Diet-induced obesity
Després, Golay, and Sjöström, 2005 [69]	Clinical study			CBR antagonist rimonabant	Rimonabant treatment decreases body weight and improves lipid profile in obese patients.	Obesity and obesity-induced disorders
Di Marzo, 2008 [15]	Review (animal/clinical studies)	CB_1_R-KO mice, obese Zucker rats	High-fat diet	CB_1_R, CB_2_R, TRPV1; CBR agonists (AEA, 2-AG), CBR antagonists (rimonabant, taranabant)	The ECS becomes dysregulated and overactivated in energy imbalance, contributing to fat accumulation and reduced adiponectin release. CB_1_R antagonists/inverse agonists improve glucose and lipid status with weight reduction in obesity and type 2 diabetes.	Obesity and related disorders
Han and Kim, 2021 [18]	Review (animal/clinical studies)			CB_1_R antagonists (2nd, 3rd generation), dual-targeting drugs (CB_1_R-CB_2_R)	CB_1_R has a crucial role in obesity-induced inflammation and in the development of metabolic syndrome. Second- and third-generation CB_1_R antagonists reduce metabolic inflammation with peripheral actions.	Obesity, obesity-induced inflammation
Scheen and Paquot, 2009 [25]	Review (clinical studies)			CB_1_R antagonists (rimonabant, taranabant)	CB_1_R blockade reduces body weight and insulin resistance, improves lipid profile and glucose tolerance, and reduces blood pressure in nondiabetic and diabetic obese patients.	Obesity, metabolic disorders

**Table 2:** Effects of endocannabinoid system and cannabinoids on carbohydrate and lipid metabolism and a relationship with insulin resistance and type 2 diabetes mellitus. Analysis from references. Original and review articles discussing CB receptor-dependent methods (KO mice, agonists, antagonists) are emphasized. CBR, cannabinoid receptors, CB_1_R and CB_2_R, cannabinoid receptors type 1 and 2, KO, knockout, 2-AG, 2-arachidonoylglycerol, THC, tetrahydrocannabinol, AEA, anandamide, ACEA, arachidonyl-2-chloroethyl amide, IR, insulin resistance, AKT, protein kinase B, GLUT2, glucose transporter 2, HFD, high-fat diet.

Ref.	Article Type	Animal Model	Diet	Receptors, Agonist/Antagonist	Short Summary	Studied Mechanism and Disease
Després et al., 2005 [69]	Clinical study			CBR antagonist rimonabant	Rimonabant treatment decreases insulin levels and improves glucose tolerance in obese patients.	Obesity, IR
Kim et al., 2011 [76]	Cells, animal study	Isolated human/ mouse islets, beta cell line, db/db and CB_1_R-KO mice		CBR agonists AEA, 2-AG, CBR antagonists AM251, AM630	Inhibition of CB_1_Rs enhances pancreatic beta cell signaling and proliferation in isolated islets, and also improves glucose tolerance and insulin sensitivity (in db/db mice).	IR
Liu et al., 2012 [77]	Cells, animal study	CB_1_R-KO mice, human/mouse hepatocytes	high-fat diet	CBR agonist anandamide	HFD induces hepatic IR in wild-type but not in CB_1_R-KO mice. CB_1_R activation contributes to diet-induced IR via hepatic CB_1_R-mediated inhibition of insulin signaling.	Obesity, IR
Shin et al., 2018 [78]	Cell/animal study	CB_1_R-KO mice, mouse beta cell line, human islet		CBR agonist 2-AG, ACEA, WIN55212, CBR antagonist AM251	CB_1_R agonists diminishes insulin secretion in β cell line and islets, whereas silencing CB_1_Rs in β cells increases expression of proinsulin, glucokinase and GLUT2 glucose transporter, which is also observed in CB_1_R-KO mice.	IR, type 2 diabetes
Motaghedi and McGraw, 2008 [79]	Cells	Adipocytes		CBR agonist 2-AG, CBR antagonist SR141716	Activation of CB_1_R by 2-AG promotes insulin sensitivity by increasing insulin-stimulated AKT phosphorylation in adipocytes, which is attenuated by CB_1_R antagonist.	IR
Hirsch and Tam, 2019 [3]	Review (animal/clinical studies)			CB_1_R antagonists	CB_1_R blockade decreases food intake and body weight, and ameliorates obesity, type 2 diabetes, fatty liver, and IR in animals. It also decreases body weight and improves glucose homeostasis in obese individuals.	Obesity, metabolic syndrome, type 2 diabetes
Di Marzo, 2008 [15]	Review (animal/clinical studies)	CB_1_R-KO mice, obese Zucker rats		CB_1_R, CB_2_R, TRPV1; CBR agonists (AEA, 2-AG), CBR antagonists (rimonabant, taranabant)	CB_1_R antagonists reduce hyperglycemia and dyslipidemia, improving insulin resistance and glucose tolerance in obesity and type 2 diabetes.	Obesity and type 2 diabetes
Nagappan, Shin, and Jung, 2019 [52]	Review (animal/clinical studies)			CB_1_R agonists/overexpression CB_1_R antagonists	CB_1_R activation modulates insulin signaling pathway and leads to insulin resistance.	Obesity, IR, type 2 diabetes

## Data Availability

Not applicable.

## Abbreviations

AEA	anandamide
2-AG	2-arachidonoylglycerol
AKT	protein kinase B
Ang II	angiotensin II
AT_1_R	type 1 angiotensin receptor
BMI	body mass index
CB	cannabinoid
CB_1_R	type 1 cannabinoid receptor
CB_2_R	type 2 cannabinoid receptor
CB_1_R-KO	type 1 cannabinoid receptor knockout (CB_1_R −/−)
CCK	cholecystokinin
CNS	central nervous system
CVD	cardiovascular disease
DAG	diacylglycerol
DAGL	diacylglycerol lipase
eCB	endocannabinoid
ECS	endocannabinoid system
FAAH	fatty acid amide hydrolase
GLUT2	glucose transporter 2
GPCR	G-protein-coupled receptor
HDL	high-density lipoprotein
HFD	high-fat diet
IR	insulin resistance
KO	knockout
LDL	low-density lipoprotein
MAG	monoacylglycerol
MAGL	monoacylglycerol lipase
NAcS	nucleus accumbens
PVN	paraventricular nucleus
T2D	type 2 diabetes mellitus
TG	triglyceride
THC	tetrahydrocannabinol
VMH	ventromedial hypothalamus
VTA	ventral tegmental area
WT	wild type
